# Cali cancer registry methods

**DOI:** 10.25100/cm.v49i1.3853

**Published:** 2018-03-30

**Authors:** Luz Stella García, Luis Eduardo Bravo, Paola Collazos, Oscar Ramírez, Edwin Carrascal, Marcela Nuñez, Nelson Portilla, Erquinovaldo Millan

**Affiliations:** 1 Registro Poblacional de Cáncer de Cali. Cali, Colombia; 2 Departamento de Patología, Facultad de Salud, Universidad del Valle, Cali, Colombia.; 3 Secretaria de Salud Pública Municipal de Cali, Cali, Colombia.; 4 Fundación POHEMA. Cali, Colombia; 5 Sistema de Vigilancia Epidemiologica de Cáncer Pediátrico (VIGICANCER), Cali, Colombia.

**Keywords:** Cancer registry, methods, data management, Cali, Colombia, Registro de cáncer, métodos, manejo de datos, Cali, Colombia

## Abstract

**Background:**

The Population Cancer Registry of Cali (RPCC) has operated since 1962, disseminating high quality information to provide a framework to assess and control the burden of cancer in Cali.

**Methods:**

The collection of new cancer cases in permanent residents of Cali is done through active search in and notification from hospitals, and public and private laboratories. The Secretary of Municipal Public Health provides individual information on general mortality and death from cancer. Tumors are coded with ICDO-3 and mortality with ICD-10. Presented rates are standardized by age and trends are assessed by estimating the percentage annual change using the regression analysis in JoinPoint. The 5-year net survival was analyzed with the Pohar-Perme estimator.

**Results:**

The 88.5% of the registered cancers had morphological verification (MV). The proportion of unknown primary site represented 5% and the death certificate only cases (DCO) varied between 0 to3% depending on the cancer site. All deaths were certified by a physician, 94.2% of cancer deaths were correctly certified. The ill-defined site proportion was 5.3% and that of uterine cancer not specified (C55) was 0.5%. For survival analysis, existing data collection procedure and infrastructure ensures assessment of the patient’s vital status and follow-up, with an average lost to follow-up of 13.2%.

**Comment:**

The information has been published in the eleven volumes of "Cancer Incidence in Five Continents" confirming high quality of the collected data. The RPCC PCRC has also participated in the Concord Study and is participating in SURVCAN-3.

## Resumen

**Antecedentes:**

El Registro Poblacional de Cáncer de Cali (RPCC) opera de manera continua desde 1962 divulgando información de calidad para proporcionar un marco para valorar y controlar el impacto del cáncer en Cali.

**Métodos:**

La recolección de los casos nuevos de cáncer en residentes permanentes de Cali se hace mediante búsqueda activa y por notificación en los hospitales y laboratorios públicos y privados. La Secretaria de Salud Pública Municipal proporciona la información individual de mortalidad general y por cáncer. Los tumores se codifican con la CIO-3 y la mortalidad con la CIE-10. Las tasas se estandarizan por edad y la tendencia se estudia con el porcentaje de cambio anual y con la regresión de JoinPoint. La supervivencia neta a 5 años se analizó con el estimador de Pohar-Perme.

**Resultados:**

El 88.5% de los canceres tuvieron verificación morfológica (VM). La proporción de los casos codificada como de sitio mal definido representó el 5% y el certificado de defunción como única evidencia del diagnóstico de cáncer (DCO) varió entre 0-3% dependiendo de la localización. Todas las defunciones fueron certificadas por un médico, el 94.2% de las muertes por cáncer fueron bien certificadas. La proporción de sitio mal definido fue 5.3% y la de cáncer de útero no especificado (C55) fue 0.5%. En todos los casos de supervivencia fue posible el seguimiento y la proporción de censuras fue 13.2%

**Comentario:**

La información ha sido publicada en los once volúmenes de “Cancer Incidence in Five Continents”. El RPCC participó en el Estudio Concord y está participando en SURVCAN-3.

## Introduction

The Population Cancer Registry of Cali (RPCC) was started in 1962 as a research program of the Department of Pathology of the Universidad del Valle. It was initially funded by a donation from the Ana Fuller Fund. Later, La Universidad del Valle became the main source of both financial and scientific resources of the registry. The RPCC began at the same time as Pan American Health Organization (PAHO) conducted the Urban Mortality Study, which examined in detail all the death certificates of the city ^1^. The systematic study of these certificates was part of the data collection for the RPCC ^2^.

Cancer registries are systems that collect information in a continuous and systematic way about each new cancer case identified within a specific population in a given area and period ^3^. There are two types of cancer registries that complement each other, although they have distinct procedures and objectives: the population-based cancer registry (PBCR) and the hospital-based Cancer Registry (HBCR). The HBCR records all cases that go to a health center or specialized service, regardless of their place of residence, for administrative and patient care purposes. The purpose of the PBCR is to identify all new cases of cancer that appear among the inhabitants of a well-defined, natural or administrative demographic area. The main objective is to produce information to provide a framework to assess and control the impact of cancer on health of the community. Some registries might be specialized on one or several tumor location(s) are called Monographic; and can be both hospital-based and population-based. Central cancer registries gather and consolidate information from several registries that cover different areas, which can also be population-based or hospital-based ^3^.

The value of the modern cancer registry and its ability to carry out cancer control activities depend to a large extent on the underlying quality of its data and the established quality control procedures ^4^. In this article, the Population-based Cancer Registry of Cali shows a standardized methodological guide and maintains the quality criteria for a reliable information system to estimate the burden of cancer in Cali.

## Obtaining new cases of cancer

### Population and registration area

**Figure 1:**
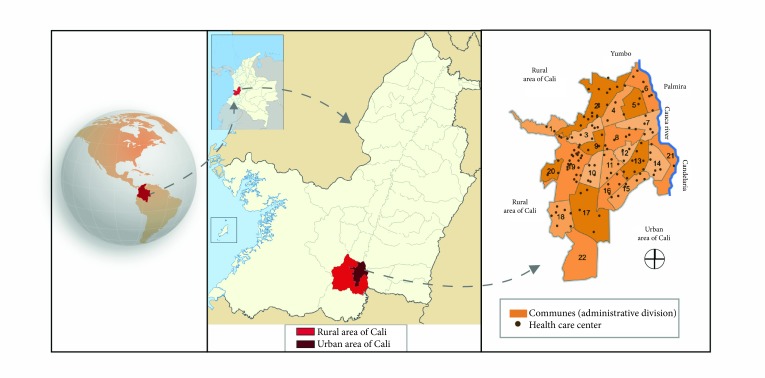
**Cali-Valle del Cauca-Colombia** geopolitical map. License: http: //creativecommons.org/licenses/by-sa/3.0/deed.es Modified by: Tejido creativo Cali-Colombia

Cali is the third largest city in Colombia, capital of the Province of Valle del Cauca, located by the Cauca river valley at coordinates 3°27'00" N 76°32'00" W. The western limit is the Farallones of Cali, which are part of the Western Cordillera of the Colombian Andes. According to both the 2005 census and National Administrative Department of Statistics of Colombia (DANE) projections, the estimated population for 2010 was 2.3 million inhabitants, 52% are women, and 26.2% self-identify as belonging to the black ethnic group ^5^ ^,^ ^6^. The life expectancy at birth was 73.1 years for men, and 78.5 years for women ^7^. The facilities for oncological care includes165 oncology services ^8^, located in the urban area, where 95% of the population resides in an area of ​​110 km^2^. This area corresponds to 20% of the extension of the municipality of Cali (561.7 km^2^) ^9^; Administratively Cali was divided into 22 communes, with a gross density of 4,094.7 inhabitants/km^2^. The rural land is approximately 424.4 km^2^ (divided into 15 corregimientos or designated areas) ^9^ with a gross density of 0.83 inhabitants/km^2^. In 2012, the municipality of Cali was defined as the cancer registry area. The geopolitical map is shown in Figure 1.

### Case definition

People of any age, residents in the urban area of ​​Cali, with a diagnosis of invasive malignant tumor for the first time (incident), of any anatomical location, that has been confirmed or treated in partial or in total. The basis for diagnosis can be both microscopic (fluid cytology, peripheral blood and bone marrow, histology of primary tumors and autopsy); and non-microscopic (clinical, surgical and imaging diagnosis). The following cancers were included: single or multiple primary malignant tumors, all tumors of the Central Nervous System and *in situ* breast and cervical cancer. Excluded are benign tumors with uncertain behavior, malignant tumors of metastatic sites, and basal cell and epidermoid carcinoma of the skin (these were included until 1986). The cases that arrived in the city for treatment or diagnosis purposes are not considered residents of Cali.

### Pediatric cancer

In 2009, an information system was setup within the RPCC for the continuous monitoring of clinical outcomes of children with cancer treated in Cali (VIGICANCER). Details have been published earlier ^10^. In summary, the system, in addition to the registry of incident cases, actively follows children under 19 years old treated in pediatric oncology units in Cali. The system includes both residents of the city and patients referred from other municipalities and departments. As part of the RPCC, it also receives information from secondary sources, achieving an exhaustiveness of around 94% and a follow-up of 95% of registered cases. The outcomes under surveillance are the vital status, relapses, abandonment of treatment and second primary cancers. This system continues to monitors patients who leave treatment and, if their vital status is unknown, they are included as events for survival analyzes. The observed survival is reported, using the Kaplan-Meier method.

## Comparability of the basic data collected

The basic information for the RPCC is collected in a pre-coded form that includes data of the person: name, sex, date of birth, age, and address. Neoplasms are described with anatomical location, morphology, behavior and, degree of differentiation, multiple primary tumors, the extent of disease (breast and cervix) and the most valid basis of cancer diagnosis. 

For the last 20 years, information on the outcomes has been collected: date of last contact, vital status, date of death, and cause of death. Neoplasms in adults are coded with ICDO-3 ^11^, whereas in children with ICCC-3 ^12^. 

To calculate date of incidence we used the guidelines of the European Network of Cancer Registries (ENCR) ^13^ and this corresponds to the date of the first histological or cytological confirmation of cancer. For the classification of multiple primary tumors, the IARC / IACR guidelines ^14^ were used, which are also used elsewhere around the world, to report the incidence rates.

## Confidentiality of information

The guidelines of the European Network of Cancer Registries (ENCR) ^13^ are followed. The director of the RPCC is responsible for the security of the information. All the staff members of the RPCC sign an agreement to guarantee the protection of the confidentiality of the data on the persons whose cancer is informed to the RPCC. Access to the physical space of the Registry is restricted to authorized persons only. The access to the confidential information is carried out using personal passwords that permit access to the computers holding the classified information and additionally closed files are used. Any data that is not used is automatically destroyed.

A single person (administrator) makes initial matching between databases to detect new cases and update vital status information. A registration number is assigned to each case and the information that identifies a patient is deleted before the data is analyzed (name and other documents that can lead to identification of the patient).

## Facilities

Universidad del Valle has been the main source of financial and technical resources. The research group at RPCC has a head quarter (287 m² area) with 15 employees working in the registry. The head of staff and his advisors are senior researchers and pathology professors at the School of Medicine. The coordinator is a business administrator with a master's degree in epidemiology and the information system is managed by an engineer with a master's degree in engineering with emphasis in systems engineering and computer science. There are three data collectors. The staff has job stability due to university affiliation that provided permanent contracts. The RPCC assures stability to the rest of the human resources using specific projects funds. The Information Technology network includes an intranet with Internet access supported by the Office of Information Technology and Telecommunications of Universidad del Valle. The local network includes a server, 11 computers and 5 laptops. Backup copies are made twice a day by means of an automatic daily script and a monthly external copy. The technical team of the RPCC meets weekly to resolve the problem cases. The software of the RPCC (Siscan) performs consistency checks when entering the data and the internal consistency is checked every six months with IarcTools ^15^. Before sending the information to international collaborators or external projects such as the IARC and the CONCORD program, the whole data set is rechecked with IarcTools ^15^.

## Periodic survey of medical specialists

The three-yearly survey of medical specialists in the city is a key activity in which several groups of students from the Faculty of Health of the Universidad del Valle have participated. This survey lasts for eight weeks and complements the continuous cancer data collection by the RPCC. As an initial step, the inventories of sites that have oncological services for the diagnosis and treatment of cancer that are not covered during routine collection activities are updated. The Faculty of Health of the Universidad del Valle is contacted, and the participating students are trained in biology, cancer nomenclature, and the methodology standardized to obtain cancer cases. Each participant is assigned a supervisor (member of the RPCC) and support materials are provided that include: 1) General recommendations; 2) minimum variables for collection; 3) list of malignant tumors; 4) manual for completing the form of the cancer morbidity survey; 5) list of assigned specialist physicians; 6) cover letters; and 7) collection forms. The supervisor has permanent contact to clarify doubts and concerns and receive weekly update of the information collected.

## Procedure for obtaining new cases of cancer

**Figure 2:**
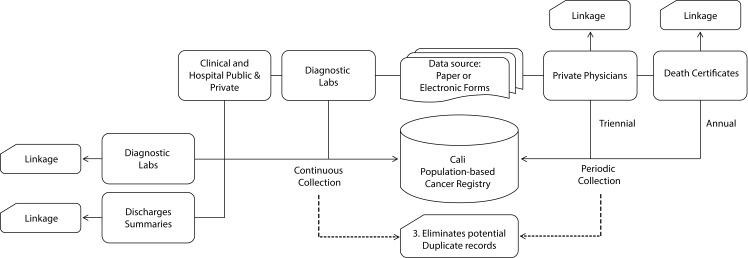
Population Cancer Registry of Cali (RPCC). Procedure to obtain new cases of cancer among permanent residents of the city through active search and notification. Collection is continuous in diagnostic laboratories, hospitals and clinics; public and private. The collection is periodic (annual and three-yearly) in the Municipal Public Health Secretariat (for death certificates), and in the physician office´s. The information is integrated into the database of the RPCC, through individual search or with matching between databases (linkage).

Figure 2 summarizes the procedures for collecting information to obtain new cancer cases among permanent residents of Cali. The information is in physical format and structured and unstructured digital formats; and the extraction of the variables of interest is done in several phases manually or automatically.

**Figure 3:**
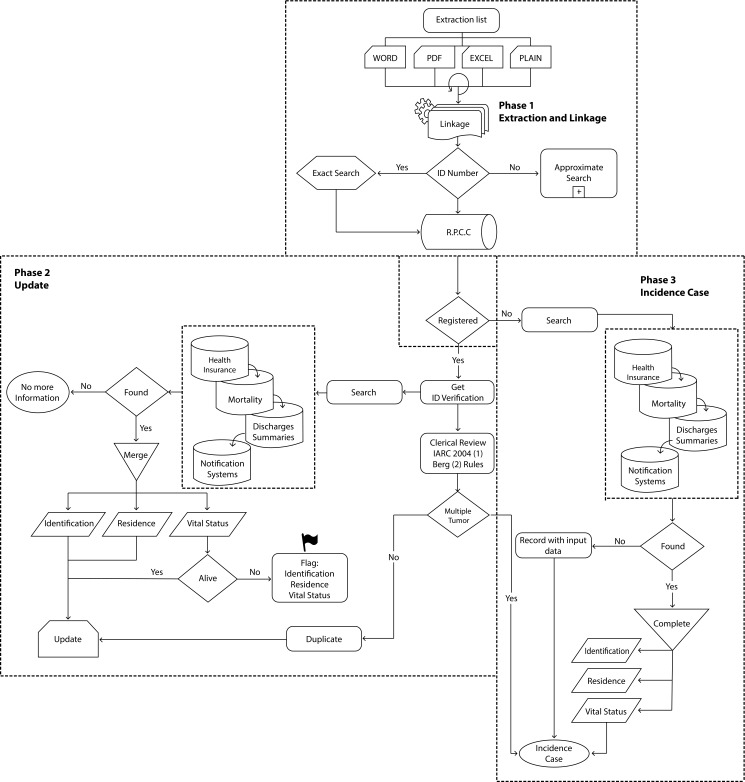
Exact search procedures (personal identity document) to detect duplicate cases, multiple tumors, update vital status, date of last contact, residence and identity of each new case of cancer.

Figure 3 shows the procedures for detection of duplicate cases, multiple tumors, updating vital status, date of last contact, residence and identity of each new case of cancer. The procedures involved three phases, which are as follow:

### Phase 1. Extraction of information

**Figure 4:**
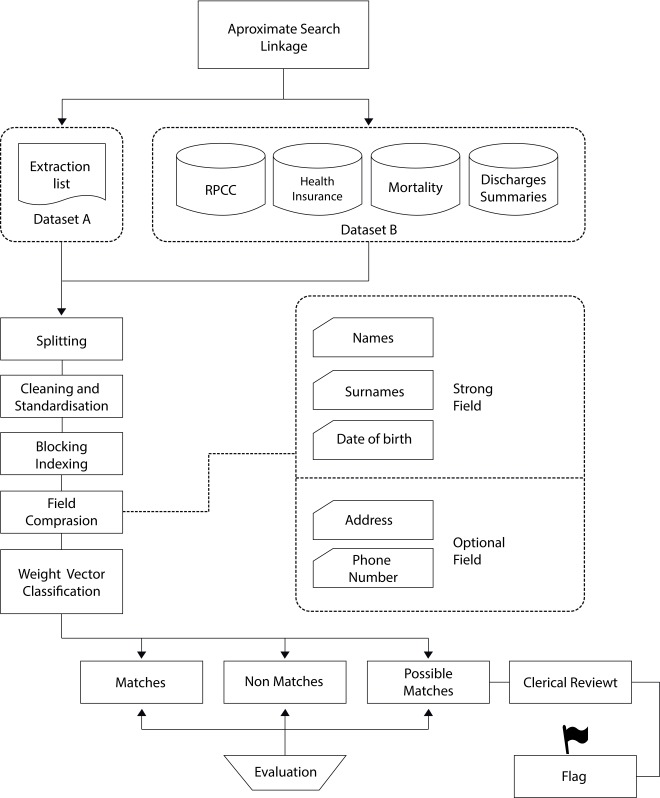
Approximate search procedures when there is no personal identity document. Pairing with the Population Cancer Registry database using the approximate search method

This is done through active search and manually when the information is in physical format and structured and unstructured digital formats; or automatic to obtain structured and unstructured listings. Hospital expenditures are obtained periodically in a structured digital format. With an automatic process of data extraction, for each case a matching with the database of the Population Cancer Registry is done in two methods: Exact search (Fig. 3) and Search by approximation (Fig. 4).

### Phase 2. Update of the information

When the cases already exist in the base of the RPCC (prevalent cancers), additional information is sought in the health insurance databases (public and private), general mortality in the city, and hospital discharges from clinics and hospitals in Cali. Information of identification, residence, date of last contact and vital state is recovered; these updated variables are marked as completed (Flag) and the case is excluded for future searches. The revised rules of the IARC 2004 ^14^ is used to allow detection of multiple primary tumors.

### Phase 3. Inclusion of new cases

In phase 3, cases that are not found in the main database of the cancer registry are processed. First, the three additional data sources are searched (Fig. 3) to find additional information that allows identification, residence and vital status to be completed. Afterwards they are entered into the main database as a new case of cancer (incidence). If additional information is not retrieved in the auxiliary databases, the case enters with only the data obtained in the extraction phase.

## Search by approximation

It is used when there is no information on the personal identification document (Fig. 4). The two sets of data to be compared are prepared namely data set (A) that are the extraction lists which contains the possible new cases of cancer and data set (B) which is the database that contains the information system of the RPCC. First the data set is divided into smaller groups to optimize matching, then standardized and indexed by blocks of similarity between two fields (names and date of birth), finally a weighted vector classification is made, where a threshold of similarity, the result is two groups of records: those that are estimated as potentially equal and those that are considered as a possible match whose process continues with a manual review, the records are evaluated to be paired between the two data sets ^16^.

## Procedures for the analysis of incidence and mortality

The International Classification of Diseases (ICD-10) ^17^ is used for the coding of cancer. The main locations were defined according to the guidelines suggested by the IARC for the analysis of the incidence information; and by the WHO to group the primary site of the tumor and the causes of (cancer) death ^18^ ^,^ ^19^. The structure of the population by sex and five-year age groups for each calendar year was obtained in the DANE ^5^. The incidence and mortality rates for the entire population were standardized by age (ASR) by means of the direct method, using as reference the world standard population ^20^ ^,^ ^21^. The global and specific rates by age and sex are expressed by 100,000 person-years. Trends in incidence rates were analyzed over ten 5-year period from 1962 to 2012; and those of mortality during six five-year periods, from 1984 to 2015. The summary measures to assess the trend of the rates over time was the annual percentage change (APC), calculated by the minimum method weighted squares ^22^. For some locations and age groups it was impossible to estimate the APC because in some years there were no new cases or cancer deaths in these categories.

## Procedure for survival analysis

### Selection criteria

Individual data from 38,671 permanent residents of Cali during the period 1995-2009, aged between 15 and 99 years, with a diagnosis of a first invasive malignant tumor in one of the following fourteen locations defined by the ICD-10 were included for the analysis. (WHO, 2012): Stomach (C16), colorectal (C18-C20), liver (C22), lung (C34), melanoma (C43), breast (C50), cervix (C53), ovary (C56), prostate (C61), thyroid (C73), Hodgkin's lymphoma (C81), non-Hodgkin's lymphoma (C82-C85, C96), multiple myeloma (C90), and leukemia (C91-C95). Following the Concord-2 study guidelines ^23^, the groups of solid tumors were defined by the anatomical site and the leukemias by their morphology. The coding of the topography and morphology was done with the International Classification of Disease for Oncology, third edition (ICD-O-3) ^11^. All malignant haematopoietic diseases were included according to the range of morphological codes of the ICD-O-3 from 9.590 to 9.999.

Excluded from the survival analysis are tumors identified as *in situ*, benign or of uncertain behavior, subjects with unknown ages, tumors detected during necropsy, cases diagnosed only through death certificate, and the syndromes myelodysplastic and myeloproliferative neoplasms such as chronic myeloid leukemia. Patients with synchronous bilateral breast cancer were included and treated as individual cases for the analysis.

## Event definition, start and end date

Death from any cause was considered an event in the survival analysis. The survival time of each case was determined by the difference in time (in days) between the date of diagnosis (index date) and the date of death, the date of last contact, or the date of the end of the study, which was defined as December 31, 2009. To compare the survival changes during the study period, the 15-year study period was divided into three: a first period between January 1, 1995 and December 31, 1999 that coincided with the implementation of the health reform in Colombia; and the other two periods; 2000-2004 and 2005-2009; after the implementation and consolidation of Law 100.

Follow-up: To update the vital status and the date of last contact, links were established between the RPCC information system and the following databases: a) general mortality of the Municipal Public Health Secretariat of Cali; b) hospital discharges from Level III institutions; c) Identification System for Potential Beneficiaries of Social Programs (SISBEN, 2016); and d) Private health insurance companies (2014). Process is described in Figures 3 and 4.

## Analysis plan

The response variable was the time between the diagnosis of cancer and the death of each individual. The maximum observation time for each subject for the failure to occur was five years. The censored variable was applied for patients who did not present the fault within the study period, and as a mechanism of censorship the loss was established during the follow-up and the end of the study. For the analysis, survival times greater than five years were censored, times after the loss to follow-up and / or as of December 31^st^, 2009. 

**Figure 5:**
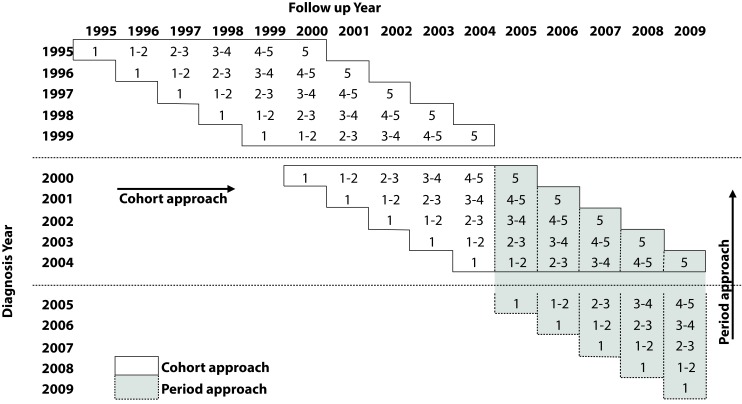
Cali Cancer Population Registry. Monitoring structure for the analysis of survival of cancer cases in permanent residents of Cali during the 1995-2009 interval with follow-up until 2009-12-31. Three 5-year periods were analyzed: 1995-1999, 2000-2004 and 2005-2009. Cohort approach between 1995-2004 and between 2005-2009. In contiguous cells the complete tracking of an interval was described. Example: All patients with a diagnosis in 1995 complete one year of follow-up in 1996, except for those diagnosed in 1995-01-01.

For the five-year periods 1995-1999 and 2000-2004, a cohort analysis was performed because all patients diagnosed with cancer during that period had at least five years of follow-up data until December 31, 2009. For the 2005-2009 period, survival analysis was carried using the period method ^24^, given that there is no complete 5-year follow-up for patients, as shown in Figure 5. The number in each cell indicates the minimum number of years of follow-up completed by patients at the end of a specific year.

For the 5-year net survival estimates, the Pohar-Perme estimator was used ^25^. Life tables of the general population of Cali in one-year age group, by sex and for each calendar year from 1995 to 2010, were provided by the Concord-2 study ^23^. Estimates of 5-year net survival were standardized by age to allow comparisons over time or with different cancer populations and with different age distributions. The three main cancer sites with similar incidence patterns by age were taken into account and the weights of the International Standard for Survival of Cancer, International Cancer Survival Standard ICSS ^26^ (Table 1) were implemented.

**Tabla 1:** International Cancer Survival Standards (ICSS) used for standardizing survival by age according to cancer site. Age classes and weighting for three types of cancer incidence age patterns

ICSS-1		ICSS-1*		ICSS-2		ICSS-3	
Group age	Weight	Group age	Weight	Group Age	Weight	Group Age	Weight
15-44	0.07	15-54	0.19	15-44	0.28	15-44	0.6
45-54	0.12	55-64	0.23	45-54	0.17	45-54	0.1
55-64	0.23	65-74	0.29	55-64	0.21	55-64	0.1
65-74	0.29	75-84	0.23	65-74	0.20	65-74	0.1
75+	0.29	85+	0.06	75+	0.14	75+	0.1
Total	1		1		1		1

ICSS-1 *: Prostate (C61)

**ICSS-1: Stomach (C16), colorectal (C18-C20), liver (C22), lung (C34), breast (C50), ovary (C53), non-Hodgkin's lymphoma (C82-C85, C96), multiple myeloma (C90), leukemia (C91-C95)**

**ICSS-2: Melanoma (C43), cervix (C53), thyroid (C73)**

**ICSS-3: Hodgkin's lymphoma (C81)**

## Exhaustiveness assessment by death certificate method

**Figure 6:**
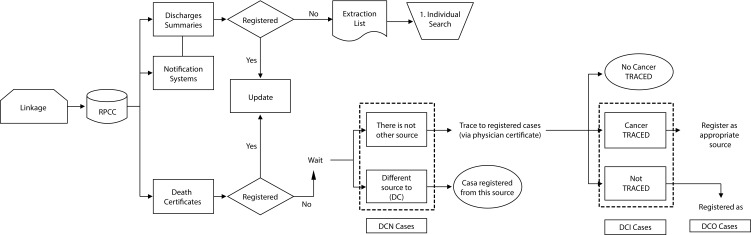
Assessing the exhaustiveness. The annual matching between the mortality database and the cancer registry makes it possible to identify new cases notified by means of the death certificate (DCN). The active and continuous search of the cases updates the most valid basis for the diagnosis and excludes some cases of mortality that are not cancer (Tracking not cancer). The remaining are the cases initiated by a death certificate (DCI) that would not have been detected by another way. After all the tracking maneuvers, there remains a residue of cases for which the only evidence of cancer was the death certificate (DCO).

To verify the exhaustiveness, the death certificate method was used ^27^. The principle is illustrated in Figure 6. Individual certificates of general mortality from all causes are received annually in a structured file in a digital format with information on causes of death in text and the basic cause codified with ICD-10 ^17^. We reviewed the causes of death to detect cancer cases that were not coded as cancer in the basic cause; and a variable is created to identify cancer cases (ICD-10: C00-C97; D05-D06, D32-D33, D45-D46, D47.1, D47.3). The initial pairing with the RPCC database allows to identify the prevalent cases that have died, the vital status and the date of death are updated. New cases reported annually through the death certificate are included in the RPCC database and are identified in a variable such as DCN. These cases will then be updated when the RPCC data collectors obtain newer information from the biopsy, the bone marrow aspirate, or the flow cytometry; the diagnostic method is updated, from death certificate to diagnosis by morphology. The active and continuous search of cases excludes some cases of mortality that are not related to cancer; and which will be used to update, once more, the diagnostic method that will convert from death certificate to diagnosis by clinical or by images. Finally, there is a remnant of cases whose only information came from death certificate (DCO). The proportion of unregistered cases that remained alive was estimated with the proportion of cases initiated by the death certificate (DCI) and the mortality: incidence ratio (M: I). Exhaustivity = $1-DCI\;*(\frac{1}{M:I})/(1-DCI)$

## Results

### Indicators of quality of the incidence information

The main quality indicators for some selected cancer sites are presented in Table 2. Age was known in 99.4% of patients. The mortality incidence ratio showed consistent values ​​except for liver (1.43) and lung (1.02). In these locations, the number of deaths was greater than the number of cases recorded in the registry.

The percentage of cases with morphological verification (MV) -histology, cytology, bone marrow aspiration and flow cytometry-, for all cancer sites was 88.5% ranging between 85-100%, except in the liver (68.3%) and lung (66.4%). In patients with leukemia, Hodgkin's lymphoma and melanoma the MV was 100%.

**Table 2:** Cali, Colombia. Indicators of the data quality of the incidence information for both sexes during the period 2008-2012

Cancer site	n	Known age (%)	M/I	1-NS	MV (%)	DCO (%)	ICD- 10
Stomach	1,810	99.7	0.78	0.83	85.1	3.0	C16
Colorectal	1,827	99.6	0.55	0.59	89.8	1.4	C18-20
Liver	467	99.8	1.43	0.95	68.3	4.5	C22
Lung	1,316	99.9	1.02	0.90	66.4	6.0	C33-34, C38-39
Skin melanoma	324	98.2	0.40	0.34	100.0	0.0	C43
Breast*	2,998	99.5	0.33	0.26	97.0	0.3	C50
Uterine cervix	1,037	99.5	0.45	0.42	94.4	1.2	C53
Ovary	513	100.0	0.59	0.66	87.1	0.6	C56
Prostate	2,937	99.2	0.32	0.17	89.2	2.0	C61
Thyroid	1,066	98.0	0.07	0.11	97.9	0.3	C73
Hodgkin's disease	154	100.0	0.20	0.36	100.0	0.0	C81
Non-Hodgkin lymphoma	1,013	99.9	0.35	0.57	99.6	0.0	C82-C85, C96
Multiple myeloma	298	99.7	0.55	0.77	99.7	0.0	C90
Leukemia	867	99.5	0.67	0.72	100.0	0.0	C91-C95
All sites	23,046	99.4	0.51		88.5	1.7	

M/I: Mortality:incidence ratio

MV: Proportion of cases verified microscopically

DCO: only evidence of death certificate

NS: Net survival

* 26 cases of breast in men are included

The percentage of cases with a death certificate only (DCO) varied between 0-3%, except in the liver (4.5%) and in the lung (6.0%). In general, for major cancer sites, they had a low percentage of cases obtained through death certificate only. Another indicator of quality that is also usually considered is the proportion of cancer cases that was coded as poorly defined site. Between the years 2008-2012 these tumors represented 4.6% of new cases of cancer in men and 5.4% in women.

### Quality indicators of survival information

During the 1995-2009 period, 40,354 cases of the selected cancers were registered, 1.73% occurred in patients under 15 years. In 2.4% there was no age information and they were excluded from the analysis. All patients had follow-up and 13.2% of the observations were censored; this proportion was higher in brain, melanoma, colorectal and ovarian cancers. In cancers with poor survival: stomach, lung, liver and pancreas; the censored rate was less than 10%. In the most frequently diagnosed cancers the censored percentage was 10.1%, 11.5% and 16.4%; for breast, prostate and cervix, respectively. In 15.3% of the cases the date of death and the date of incidence were the same.

### Quality indicators of cancer mortality certification

Mortality due to cancer represented 18.0% (23,793 / 132,397) of the total deaths that occurred in the city during the period 2006-2015. 0.8% of the cases were not coded as cancer in the basic cause. All deaths were certified by a physician; the proportion of poorly defined site (C76-C80, C97) was 5.3% and that of the uterine cancer not specified (C55) was 0.5%. Only 4 (0.02%) of the death certificate cases did not have age information. 94.2% of cancer deaths were well certified.

All patients died from cancer during the 2008-2012 period were found in the cancer registry database. For recognized sites of metastasis; liver, lung, bone and brain; the ICD-10 (17) code of the death certificate was compared with the topographic code of the ICD-0-3 (11) assigned by the cancer registry. Table 3 shows the concordance of the two systems to assign the code for each of the described locations. 45% of the deaths coded as liver cancer in the death certificate corresponded to metastasis. In the RPCC there was evidence (morphological and clinical verification) of having made the diagnosis of cancer in the patient's life in a different primary site. This proportion reached values ​​of 46%, 15% and 10% for bone, lung and CNS locations.

**Table 3:** Cali, Colombia. Coding concordance for some selected sites between the Cali Population Registry of Cali and the Municipal Public Health Secretariat. 2008-2012.

Information source			Cancer registry				
Municipal Public Health Secretary	Location		Liver				
			Yes	No	Kappa	IC 95%	
	Liver	Yes	341	284	0.6382	0.60	0.68
		No	3	1,819			
	Lung		Lung				
		Yes	980	250	0.7902	0.75	0.83
		No	7	1,210			
	Bone		Bone				
		Yes	50	43	0.6711	0.63	0.71
		No	4	2,350			
	SNC		SNC				
		Yes	430	69	0.8985	0.50	1.29
		No	8	1,940			

IC 95%: confidence interval 95%

Kappa: Concordance: <0.00: no agreement; 0.00-0.20: slight; 0.21-0.40: fair; 0.41-0.60: moderate; 0.61-0.80: substantial; 0.81-1.00: almost perfect

### Exhaustiveness assessment by death certificate method

The 54% of people who died from cancer in Cali during the 2008-2012 period were already included in the RPCC database. The new cases notified by means of the death certificate (DCN) corresponded to 46% of the deaths of the period. The proportion was higher in cancers with high lethality as stomach (45%); and lower, in patients with breast cancer (12%), cervix (18%), prostate (14%) and childhood cancer (23%), Table 4. The exhaustivity index was greater than 90%, except in cases of prostate cancer.

**Table 4:** Population Registry of Cancer of Cali, Colombia. Percentage of registered cases as DCN, DCO, and mortality ratio: incidence; in selected locations. Period 2008-2012.

Cancer site	DCN	DCN/M	DCO	DCO/M	Incidence	mortality	M:I	DCI	DCI/M	Exhaustiveness
	n	%	n	%	n	n		n	%	
All	5,327	0.46	371	0.03	23,046	11,664	0.51	1,403	0.12	0.87
Stomach	625	0.45	53	0.04	1,810	1,374	0.72	99	0.07	0.97
Colorectal	332	0.34	25	0.03	1,827	987	0.53	68	0.07	0.94
Breast	115	0.12	7	0.01	2,972	941	0.32	32	0.03	0.92
Cervix	81	0.18	11	0.02	1,037	462	0.45	16	0.03	0.96
Prostate	126	0.14	54	0.06	2,937	913	0.31	103	0.11	0.72
Child <15	38	0.23	1	0.01	402	167	0.42	3	0.018	0.97

M:I Mortality:Incidence ratio

DCN: New cases notified annually to the RPCC through the death certificate.

DCI: DCN - cases excluded from mortality that are not cancer

DCO: New cases in the RPCC whose only evidence of cancer is the death certificate.

exhaustiveness: (1-DCI * (1 / M: I)) / (1-DCI)

### Periodic survey of oncological services

The last survey conducted in 2014 costed US $ 23,300, where 19 students participated, each one received a bonus, transportation cost and one payment per survey. During the eight weeks, 107 oncological services were visited, located in medical centers (64), private clinics (36) and private offices (5). Information was obtained that identified 2,215 new cases of cancer (27.8%) and updated 5,750 cases (72.2%) that were already in the database of the RPCC.

## Discussion

The Cali cancer registry is the only one registry in low and middle income countries that has accurately reported the cancer situation continuously over the last half century. The information is of high quality and has been included in all eleven volumes of Cancer Incidence in Five Continents (CI5) ^21^ ^,^ ^28^ ^-^ ^37^. 

For forty years, RPC-Cali and was the only valid source of information on the incidence of cancer in Colombia ^2^. The National Cancer Institute of Colombia (INC-Col) with the support of Universidad del Valle, promoted in the first decade of the 21st century the establishment of RPCs in strategic regions of the country to increase coverage. Due to this effort, the incidence information of the Colombian cities of Pasto, Manizales and Bucaramanga was added to that of Cali and published since 2012 in CI5 ^36^, and the four Colombian RPCs participated in the CONCORD study ^23^, the global program for global surveillance of cancer survival, led by the London School of Hygiene and Tropical Medicine. 

Currently, the RPC-Cali participates in SURVCAN-3, an initiative of the IARC to produce reliable and comparable survival statistics for countries in transition. Due to the great strength of the Cancer Registry, Cali is the first city in the world to implement the initiative "C/Can 2025: Challenge of Cities Against Cancer"; an initiative of the International Union for Cancer Control (UICC) that seeks to increase the coverage and quality of oncological care in the cities of more than one million inhabitants of low and middle income countries.

### Success factors of the RPCC

Several factors have contributed to the stability and continuity over time of the Cali Cancer Registry. The RPCC has standardized definitions and procedures for the collection, analysis, storage, validation and dissemination of information. Universidad del Valle has been the main source of financial and technical resources. The four directors that the RPCC has had in the 55 years of operation have been academics and researchers of the Department of Pathology, in charge of coordinating a trained human resource that belongs to the plant of the Universidad del Valle. The RPCC is constituted as a research group and ranked at the top of the Colombian research system. With specific projects, it provides solution to epidemiological problems and complements information gathering activities. The total cost per case in the RPCC was US $82, which included US $ 25 for fixed-cost activities, US $ 43 for central variable-cost activities, and US $ 14 for other activities ^38^.

The RPCC has social recognition in the city, thus facilitating the process of data collection that is made passively and actively from the various sources of data information. The oncological care facilities in Cali, include 165 oncology services enabled ^8^ to offer accurate diagnosis and adequate treatment to 9,000 patients per year ^39^. Since its foundation in 1962, the RPCC limited the registration area to the urban area of ​​Cali and developed a clear definition of "case", including only the new cases of cancer diagnosed in the permanent residents of the city; and excluding the cases of patients referred to the city for diagnostic and / or treatment procedures. 

To estimate the rates and to construct the life tables for the survival study, reliable denominators based on population censuses and projections are required. The DANE facilitated the demographic structure of the population for the period 1962-2015.

### Regulations for the notification of cancer in Colombia.

The Colombian government positioned cancer as a primary public health problem and established actions for comprehensive care to reduce morbidity and mortality due to this disease and improve the quality of life of cancer patients. Surveillance and control mechanisms were implemented, and the National Cancer Information System was organized. The model of care for these diseases was defined in the Ten-Year Plan for Cancer Control in Colombia 2012-2021 ^40^, and the axis of this strategic plan is surveillance, situational analysis and research. The Ministry of Health and Protection of Colombia (Ministerio de Salud y Protección Social) regulated the basic data that health insurers and health entities must report on the oncological services provided, whether they are promotion, prevention, diagnosis, treatment or rehabilitation (RIPS). For the management of information on public health, the Public Health Surveillance System (SIVIGILA) was regulated for the surveillance of breast cancer, cervical cancer and childhood cancer. The objective is to determine the opportunity at the beginning of the treatment of confirmed cases and to estimate the frequency of cases detected at different stages. Since 2014, health insurers (EPS) must report cancer information to the High Cost Account (CAC), a non-governmental organization that was created to guide the management of health risk and ensure the management of the disease of the people affected.

### Strengths of the RPCC: Quality indices during the period 2008-2012

The value of a cancer registry depends greatly on the quality of the data and on the quality control procedures in force ^4^. The RPC-Cali takes four dimensions into account to determine the quality indicators of the data collected: comparability, validity, timeliness and exhaustiveness.

#### Comparability

The RPCC uses standard methods to make the information comparable to other regions of the country and the world. The neoplasms are coded with the ICD-O-3 for adults ^11^ and the ICCC-3 for children ^12^. For date of incidence, the guidelines of the ENCR (13) are followed and the IARC guidelines for the classification of multiple primary tumors were used ^14^.

#### Validity

The main and most reliable sources of data for the cancer registry are the histopathology reports; but they are not enough to guarantee clarity, such as poorly accessible tumors: those of the CNS, pancreas, lung, retroperitoneum and others; the basis of the diagnosis can be imaging studies, clinical examination and DCO.

The percentage of RPC-Cali cases with a morphologically verified diagnosis (MV%) was 88.5%, similar to other RPC-Colombians and RPC-Latin American; and inferior to the majority of PRC-Europeans and North American RPCCs (90% -95%) ^37^. Africa has the two contrasts (53.9% Uganda: Kyandono Country, 97.8% Algeria: Sétif) ^37^. In low and middle income countries, a large proportion of cases diagnosed through the pathology service may suggest deficiencies in the search for cases and, therefore, evidence of incomplete registration.

In the RPC-Cali, the percentage of cases known only by death certificate (DCO%) was 1.7%; the lowest of all the RPC-Latin American; and like most RPC-North American and RPC-European ^37^. Some RPCs in Africa and Latin American have DCO% greater than 10%; which indicate poor case detection and poor quality, because death certificates do not provide information on the morphology of the tumor. A high proportion of new cases of cancer based on a clinical diagnosis has the same interpretation.

#### Exhaustiveness

The incidence rates have been stable over time and the expected values ​​are comparable with those reported by cancer registries that serve similar populations such as Quito (192.8 person-years and 198.9 person-years in men and women, respectively) and Costa Rica (173.9 person-years and 167.0 person-years in men and women, respectively) ^37^. 

The collaborative work with the SSPM of Cali facilitates access to information on general mortality and cancer; and allows us to have an independent source of verification of new cases of cancer. Cancer deaths were well certified at about 94.2%. The M:I ratio for all cancer sites during the period 2008-2012 was 51%; similar to that of other RPC-Latin American (range, 38.3% to 68%) ^36^ and higher than that reported by the United States (34.8% in men, and 36% in women) ^36^ through the SEER (Surveillance, Epidemiology, and End Results Program). In many Latin American countries, the M:I ratio is greater than one in tumors with high fatality such as pancreas, liver, esophagus. Fatality of these cancers are due to lack of complete information and/or lack of diagnosis when the patient was alive.

The exhaustivity index was 87% (method of death certificates IE-CD) and in the cancers prioritized by the PNDC it was greater than 90%, except in cases of prostate cancer (72%). This RPC-Cali index is higher than that reported by other international cancer registers (82.8% in Japan, Miyagi, 80.4% in Germany, Münster and 65.6% in the United Kingdom) ^41^. 

The method depends on the availability of relatively good quality certificates, which mention the cause of death (completely and accurately) in the area covered by the cancer registry. This method has not been applied in other RPC-Latin American countries.

#### Opportunity

The statistics of the cancer situation in Cali are public access after 36 months following the year of diagnosis. Data is also available on the RPPC portal http://rpcc.univalle.edu.co. This information describes 50 years of incidence (1962-2012), 30 years of mortality (1984-2014) and 15 years of survival (1995-2009).

### Limitations

The data of each service in each institution are handled autonomously and independently. because the information is managed on different platforms, generating duplication of data, data transfer difficulties and a decrease in the quality and integrity of the information. 

The Colombian oncology services periodically notify to different dependencies of the ministry of health (SIVIGILA, RIPS, CAC). These legacy systems are mostly local applications that lack interoperability for proper data management. Institutions begin to perceive notification as a burden and relegate them and deprioritize data transfer to the cancer registry. This complexity is a risk factor to guarantee completeness in the collection of information. Consequently, there are great possibilities of underestimating the cancer risk in the population. It is urgent to modify the current Ministry of Health regulations so that the RPC-Colombians are incorporated into the cancer information system with an adequate budget allocation.

### Future challenges

The implementation of standards and transfer mechanism, shared information flows and adoption of tools are priorities to communicate effectively with different information systems in the city of Cali. Also set public policies that facilitate the implementation of these solutions. And then, the creation of an interinstitutional data warehouse is essential to provide key support for making decisions both public at the population level and administrative at the clinical level. The main objective of this implementation is to guarantee quality information for knowledge management proposes.

## References

[1] Puffer RR, Grifith GW (1968). Características de la Mortalidad Urbana. Informe de la investigación interamericana de mortalidad.

[2] Correa P (2012). The Cali cancer registry an example for latin america. Colomb Med (Cali).

[3] dos Santos-Silva I (1999). Cancer Epidemiology: principles and methods. Lyon:.

[4] Bray F, Parkin DM (2009). Evaluation of data quality in the cancer registry principles and methods. Part I: comparability, validity and timeliness. European J Cancer.

[5] (2010). Departamento Administrativo Nacional de Estadística Estimaciones y proyecciones de población periodo 1985-2020.

[6] Krystosik AR, Curtis A, Buritica P, Ajayakumar J, Squires R, Dávalos D (2017). Community context and sub-neighborhood scale detail to explain dengue, chikungunya and Zika patterns in Cali, Colombia. PLoS ONE.

[7] Pan American Health Organization (2012). Health in the Americas: 2012 Edition. Regional Outlook and Country Profiles. Washington,.

[8] Murcia E, Aguilera J, Wiesner C, Pardo C (2018). Oncology services supply in Colombia. Colomb Med (Cali).

[9] Departamento Administrativo de Planeación (2015). Cali en Cifras.

[10] Ramirez O, Aristizabal P, Zaidi A, Ribeiro RC, Bravo LE (2018). Implementing a childhood cancer outcomes surveillance system within a population-based cancer registry. J Global Oncol.

[11] Percy C, Fritz A, Jack A, Shanmugarathan S, Sobin L, Parkin DM (2000). International classification of diseases for oncology (ICD-O-3).

[12] Steliarova-Foucher E, Stiller C, Lacour B, Kaatsch P (2005). International classification of childhood cancer. Cancer.

[13] Tyczynski JE, Démaret E, Parkin DM (2003). Standards and guidelines for cancer registration in Europe: The ENCR recommendations. Vol 1.

[14] Working Group Report (2005). International Rules for Multiple Primary Cancers (ICD-O third Edition). Eur J Cancer Prev.

[15] Ferlay J, Burkhard C, Whelan S, Parkin DM (2005). Check and conversion programs for cancer registries (IARC/IACR Tools for Cancer Registries).

[16] Christen P Febrl - A freely available record linkage system with a graphical user interface.

[17] WHO (2004). International statistical classification of diseases and related health problems, 10th revision (ICD-10).

[18] Bray F, Ferlay J, Laversanne M, Brewster DH, Gombe Mbalawa C, Kohler B (2015). Cancer incidence in five continents inclusion criteria, highlights from Volume X and the global status of cancer registration. Int J Cancer.

[19] WHO (2017). Methods and data sources for country-level causes of death 2000-2015.

[20] Segi M (1960). Cancer mortality for selected sites in 24 countries (1950-57).

[21] Doll R, Payne P, Waterhouse JAH (1966). Cancer Incidence in Five Continents Vol I.

[22] National Cancer Institute, Surveillance Research Program SEER*Stat software.

[23] Allemani C, Weir HK, Carreira H, Harewood R, Spika D, Wang XS (2015). Global surveillance of cancer survival 1995-2009: analysis of individual data for 25,676,887 patients from 279 population-based registries in 67 countries (CONCORD-2). Lancet.

[24] Brenner H, Gefeller O (1997). Deriving more up-to-date estimates of long-term patient survival. J Clin Epidemiol.

[25] Perme MP, Stare J, Estève J (2012). On estimation in relative survival. Biometrics.

[26] Corazziari I, Quinn M, Capocaccia R (2004). Standard cancer patient population for age standardising survival ratios. Eur J Cancer.

[27] Parkin DM, Bray F (2009). Evaluation of data quality in the cancer registry principles and methods Part II. Completeness. Eur J Cancer.

[28] Doll R, Muir CS, Waterhouse JAH (1970). Cancer Incidence in Five Continents.

[29] Waterhouse J, Muir CS, Correa P, Powell J (1976). Cancer Incidence in Five Continents.

[30] Waterhouse J, Muir CS, Shanmugaratnam K, Powell J (1982). Cancer Incidence in Five Continents.

[31] Muir CS, Waterhouse J, Mack T, Powell J, Whelan SL (1987). Cancer Incidence in Five Continents.

[32] Parkin DM, Muir CS, Whelan SL, Gao YT, Ferlay J, Powell J (1992). Cancer Incidence in Five Continents.

[33] Parkin DM, Whelan SL, Ferlay J, Raymond L, Young J (1997). Cancer Incidence in Five Continents.

[34] Parkin DM, Whelan SL, Ferlay J, Teppo L, Thomas DB (2002). Cancer Incidence in Five Continents.

[35] Curado MP, Edwards B, Shin HR, Storm H, Ferlay J, Heanue M (2007). Cancer Incidence in Five Continents.

[36] Forman D, Bray F, Brewster DH, Gombe MC, Kohler B, Piñeros M (2014). Cancer Incidence in Five Continents.

[37] Bray F, Colombet M, Mery L, Piñeros M, Znaor A, Zanetti R (2017). Cancer Incidence in Five Continents.

[38] de Vries E, Pardo C, Arias N, Bravo LE, Navarro E, Uribe C (2016). Estimating the cost of operating cancer registries Experience in Colombia. Cancer Epidemiol.

[39] Bravo LE, Arboleda O, Ramirez O, Durán A, Lesmes MC, Rendler-García M (2017). Cali, Colombia, Key learning City C/Can 2025: City Cancer Challenge. Colomb Med (Cali).

[40] Plan Decenal para el Control del Cáncer en Colombia.2012 - 2021 (2012). Ministerio de Salud y Protección Social.

[41] Bray F, Parkin DM (2009). Evaluation of data quality in the cancer registry: principles and methods. Part II: Completeness. European J Cancer.

