# Cali Cancer Registry Methods

**DOI:** 10.25100/cm.v49i2.4056

**Published:** 2018-06-30

In this article by Garcia et al, On corresponding author is incorrect: “Luz Stella Garcia, Registro Poblacional de Cancer de Cali. Calle 4B 31-00 Oficina 4003, Edificio 116, Cali, Colombia. E-mail: luz.garcia@correounivalle. edu.co.” The correct corresponding author should be: “Luis Eduardo Bravo, Director Registro Poblacional de Cáncer de Cali. Calle 4B 31-00 Oficina 4003, Edificio 116, Cali, Colombia. E-mail: luis.bravo@correounivalle.edu.co”

