# Supplementary material for: Cali cancer registry methods
Source: Colomb Med (Cali). 2018 Mar 30;49(1):109–20. doi: 10.25100/cm.v49i1.3853 (PMC6018820; doi:10.25100/cm.v49i1.3853)
Supplement: Related texts

# Research article: Métodos del Registro de Cáncer en Cali, Colombia

## Introducción

El Registro Poblacional de Cáncer de Cali (RPCC) se inició en 1962 como un programa de investigación del Departamento de Patología de la Universidad del Valle, fue inicialmente financiado por una donación del Fondo Ana Fuller, posteriormente, la Universidad del Valle ha sido la principal fuente de recursos financieros y de personal científico y técnico del registro. El RPCC comenzó al mismo tiempo que la Organización Panamericana de la Salud realizó el Estudio de Mortalidad Urbana, que examinó en detalle todos los certificados de defunción donde se incluyeron los de la ciudad Cali ^1^. El estudio sistemático de dichos certificados fue parte de la recopilación de datos para el RPCC ^2^.

Los registros de cáncer son sistemas que recopilan información de manera continua y sistemática sobre cada caso nuevo de cáncer identificado dentro de una población específica en un periodo de tiempo determinado ^3^. Hay dos clases de registros de cáncer que se complementan mutuamente, aunque tienen funcionamiento y objetivos parcialmente diferentes: el registro de cáncer de base poblacional (RCBP) y el Registro de Cáncer Hospitalario (RCH). El RCH contempla todos los casos que acuden a un centro sanitario o servicio especializado, independientemente de su lugar de residencia, tiene fines administrativos y de atención al paciente. El RCBP tiene como finalidad identificar todos los casos nuevos de cáncer, que aparecen entre los habitantes de un área demográfica bien definida, natural o administrativa. El principal objetivo es producir información para proporcionar un marco para valorar y controlar el impacto del cáncer en la comunidad. Algunos registros que son selectivos y solo estudian una localización tumoral se denominan Monográficos; y pueden ser tanto de base hospitalaria como poblacional. Los registros centrales de cáncer reúnen y consolidan la información de varios registros que cubren áreas diferentes, también pueden ser de base poblacional u hospitalaria ^3^. 

El valor del registro de cáncer moderno y su capacidad para llevar a cabo actividades de control del cáncer dependen en gran medida de la calidad subyacente de sus datos y los procedimientos de control de calidad establecidos ^4^. En este artículo, el Registro Poblacional de Cáncer de Cali presenta una guía metodológica estandarizada conservando los criterios de calidad para los registros de cáncer que permite mantener un sistema de información fiable para realizar la estimación de indicadores del impacto del cáncer en Cali.

## Obtención de los casos nuevos de cáncer

### Población y área de registro

Cali es la tercera ciudad de Colombia, capital del Departamento del Valle del Cauca, ubicada en el valle del rio Cauca en las coordenadas 3°27′00″N 76°32′00″O. El límite occidental son los Farallones de Cali, que hacen parte de la Cordillera Occidental de los Andes Colombianos. Según el censo del 2005 y de acuerdo con las proyecciones del DANE, la población estimada para el 2010 fue 2.3 millones de habitantes, 52% son mujeres, y 26.2% se auto reconocen como pertenecientes a la etnia negra ^5^^,^^6^. La expectativa de vida al nacimiento es 73.1 años para hombres, y 78.5 años para las mujeres ^7^. La infraestructura para la atención oncológica cuenta con 165 servicios oncológicos habilitados ^8^, ubicados en el área urbana, donde reside el 95% de la población en un área de 110 km que corresponde al 20% de la extensión del municipio de Cali (561.7 km^2^) ^9^. 

Desde su fundación, el área de registro ha sido la zona urbana de Cali, dividida administrativamente en 22 comunas, con una densidad bruta de 184.7 habitantes/km^2^. El suelo rural es de 424.4 km^2^ (dividido en 15 corregimientos) ^9^ con una densidad bruta de 0.83 habitantes/ km^2^. En el 2012 se definió como área del registro de cáncer el municipio de Cali. El mapa geopolítico se muestra en la Figura 1. 

**Figura 1:** Mapa geopolítico Cali-Valle del Cauca-Colombia. Licencia:http://creativecommons.org/licenses/by-sa/3.0/deed.es Modificado por: Tejido creativo Cali-Colombia

### Definición de caso

Hombres y mujeres de cualquier edad, residentes en el área urbana de Cali, con diagnóstico de tumor maligno invasivo por primera vez (incidente), de cualquier localización anatómica, independiente que haya sido confirmado o tratado en forma parcial o total. La base para el diagnóstico puede ser tanto microscópica (citología de líquidos, sangre periférica y médula ósea, histología de tumores primarios y autopsia); como no microscópica (diagnóstico clínico, quirúrgico e imagenológico). Se incluyen tumores malignos primarios únicos o múltiples, todos los tumores del Sistema Nervioso Central y cáncer *in situ* de mama y cérvix. Se excluyen tumores benignos y de comportamiento incierto, tumores malignos de sitio metastásico y carcinoma basocelular y epidermoide de piel (fueron incluidos hasta el año 1986). Los casos que han llegado a la ciudad para fines de tratamiento o diagnóstico no son considerados residentes de Cali. 

### Cáncer infantil

En el 2009 se implementó dentro del RPCC un sistema de información para el monitoreo continuo de los resultados clínicos de niños con cáncer tratados en Cali (VIGICANCER), cuyos detalles ya han sido publicados ^10^. En resumen, el sistema además del registro de casos incidentes hace seguimiento activo a los menores de 19 años tratados en Cali en unidades de oncología pediátrica. El sistema incluye tanto residentes de la ciudad como pacientes remitidos de otros municipios y departamentos. Al ser parte del RPCC, también recibe información por fuentes secundarias, logrando una exhaustividad de alrededor del 94% y un seguimiento del 95% de los casos registrados. El estado vital, la recaída de la enfermedad, el abandono del tratamiento y las segundas neoplasias son los desenlaces bajo vigilancia. El sistema continúa el seguimiento de los pacientes que abandonan tratamiento y en caso de no conocerse su estado vital se incluyen como eventos para los análisis de supervivencia. Se reporta la supervivencia observada, utilizando el método de Kaplan-Meier.

### Comparabilidad de los datos básicos recolectados

La información básica para el RPCC se recolecta en un formulario precodificado que incluye datos de la persona: nombre, sexo, fecha de nacimiento, edad, dirección. Las neoplasias se describen con la localización anatómica, la morfología, el comportamiento, el grado de diferenciación, los tumores múltiples primarios, la extensión de la enfermedad (mama y cérvix) y la base más valida del diagnóstico de cáncer. Desde hace 20 años se obtiene información del desenlace: fecha de último contacto, estado vital, fecha de defunción, causa de defunción. En los adultos las neoplasias se codifican con la CIEO-3 ^11^ y en los niños con la ICCC-3 ^12^. Para la fecha de incidencia se siguen las guías de la *European Network of Cancer Registries (ENCR)*^13^ y corresponde a la fecha de la primera confirmación histológica o citológica del cáncer. Para la clasificación de los tumores primarios múltiples se siguen las guías de IARC/IACR ^14^ que son utilizadas en el resto del mundo, para informar las tasas de incidencia.

### Confidencialidad de la información

Se siguen las guías de la *European Network of Cancer Registries (ENCR)* ^13^. El director del RPCC es responsable de la seguridad de la información. Todos los integrantes del RPCC firman un acuerdo para garantizar la protección de la confidencialidad de los datos sobre las personas cuyo cáncer se informa al RPCC. El acceso al espacio físico del Registro está restringido a las personas autorizadas. Hay control de la información considerada confidencial mediante claves de acceso a los ordenadores, archivos cerrados y destrucción de material con identificación cuando ésta ha dejado de ser de utilidad. Una sola persona (administrador) hace los apareamientos iniciales entre bases de datos para detectar casos nuevos y actualizar la información de estado vital y último contacto. A cada caso se le asigna un número de registro y la información que identifica a un paciente se elimina cuando se analizan los datos (nombre y documento de identidad). 

### Infraestructura

La Universidad del Valle ha sido la principal fuente de recursos financieros y técnicos. Las oficinas del RPCC (área de 287 m²) alojan a 15 personas que trabajan en el registro. El director y el exdirector quien trabaja como asesor son investigadores senior y profesores de Patología en la Escuela de Medicina. La coordinadora es administradora de empresas con maestría en epidemiologia y el sistema de información lo administra una ingeniera con maestría en ingeniería con énfasis en ingeniería de sistemas y computación. Hay tres recolectores de planta. El personal tiene estabilidad laboral porque todos están vinculados a la universidad mediante contratos a término indefinido. El RPCC es un grupo de investigación y mediante proyectos específicos financia el resto del talento humano. La infraestructura de TI incluye una intranet con acceso a Internet respaldada por la Oficina de Tecnología de la Información y Telecomunicaciones de la Universidad del Valle. La infraestructura local incluye un servidor, 11 computadoras y 5 computadoras portátiles. Las copias de seguridad se realizan dos veces al día por medio de un guion diario automático y una copia externa mensual. El equipo técnico del RPCC se reúne semanalmente para resolver los casos problema. El software del RPCC (Siscan) efectúa controles de coherencia al ingresar los datos y semestralmente se verifica la consistencia interna con IarcTools ^15^. Antes de enviar la información a IARC y al programa CONCORD, se chequea una vez más todo el conjunto de datos con IarcTools ^15^

### Encuesta periódica a médicos especialistas

La encuesta trienal a médicos especialistas de la ciudad es una actividad muy importante en la que han participado varias cohortes de estudiantes de la Facultad de Salud de la Universidad del Valle. Esta encuesta tiene una duración de ocho semanas y complementa la recolección continua que hace el RPCC. Como paso inicial se actualiza el censo de los sitios que tienen servicios oncológicos para el diagnóstico y tratamiento de cáncer que no son cubiertos durante las actividades de recolección rutinaria. Se convoca a la Facultad de Salud de la Universidad del Valle y a los estudiantes que acuden se capacitan sobre biología, nomenclatura del cáncer, y la metodología estandarizada por el registro de cáncer para la obtención de los casos de cáncer. A cada participante se le asigna un supervisor (integrante del RPCC) y se entrega material de apoyo que incluye: 1) Recomendaciones generales; 2) variables mínimas a recolectar; 3) listado de tumores malignos; 4) manual para el diligenciamiento del formulario de la encuesta de morbilidad de cáncer; 5) lista de médicos especialistas asignados; 6) cartas de presentación; 7) formularios de recolección. El supervisor tiene contacto permanente para aclarar las dudas e inquietudes y recibir semanalmente la información recolectada. 

### Procedimiento para obtención de casos nuevos de cáncer 

La Figura 2 presenta de manera resumida los procedimientos de recolección de la información para obtener los casos nuevos de cáncer en residentes permanentes dentro de los límites de Cali. La información se encuentra en formato físico y formatos digitales estructurados y no estructurados; y la extracción de las variables de interés se hace en varias fases de manera manual o automatizada.

**Figura 2:** Registro Poblacional de Cáncer de Cali (RPCC). Procedimiento para obtener los casos nuevos de cáncer en residentes permanentes de la ciudad mediante búsqueda activa y notificación. La recolección es continua en los laboratorios diagnósticos, hospitales y clínicas; públicas y privadas. La recolección es periódica (anual y trienal) en la Secretaria de Salud Pública Municipal (certificados de defunción), y en los consultorios médicos. La información se integra en la base de datos del RPCC, mediante búsqueda individual o con apareamiento entre bases de datos (linkage)

La Figura 3 muestra los procedimientos para detectar duplicados, tumores múltiples, actualizar el estado vital, fecha de último contacto, residencia e identidad de cada caso nuevo de cáncer. 

**Figura 3:** Procedimientos de búsqueda exacta (documento de identidad personal) para detectar duplicados, tumores múltiples, actualizar el estado vital, la fecha de último contacto, la residencia y la identidad de cada caso nuevo de cáncer

#### Fase 1. Extracción de la información

Se hace mediante búsqueda activa y de manera manual cuando la información se encuentra en formato físico y formatos digitales estructurados y no estructurados; o automática para obtener listados estructurados y no estructurados. Los egresos hospitalarios se obtienen de manera periódica en formato digital estructurado. Con un proceso automático de extracción de datos, para cada caso se realiza un apareamiento con la base de datos del Registro Poblacional de Cáncer en dos métodos: Búsqueda exacta (Fig. 3) y Búsqueda por aproximación (Fig. 4). 

**Figura 4:** Procedimientos de búsqueda por aproximación cuando no hay documento de identidad personal. Apareamiento con la base de datos del Registro Poblacional de Cáncer usando el método de búsqueda por aproximación.

#### Fase 2. Actualización de la información

Cuando los casos ya existen en la base del RPCC (cánceres prevalentes), se busca información adicional en las bases de seguros de salud (públicas y privadas), mortalidad general de la ciudad; y egresos hospitalarios de clínicas y hospitales de Cali. Se recupera información de identificación, residencia, fecha de último contacto y estado vital; éstas variables actualizadas quedan señaladas como terminadas (Flag) y el caso se excluye para búsquedas futuras. La revisión de las reglas de la IARC 2004 ^14^ permite detectar los tumores primarios múltiples. 

#### Fase 3. Inclusión de los casos nuevos

En la fase 3 se procesan los casos que no se encuentran en la base principal del registro de cáncer. En primer lugar, se buscan en las tres fuentes auxiliares (Figura 3) para encontrar información adicional que permite completar identificación, residencia y estado vital, luego se ingresan a la base principal como un nuevo caso de cáncer (incidencia). Si no se recupera información adicional en las bases auxiliares, el caso ingresa con solo los datos obtenidos en la fase de extracción. 

#### Búsqueda por aproximación

Se utiliza cuando no hay información del documento de identificación personal, (Figura 4). Se preparan los dos conjuntos de datos a comparar; en el conjunto de datos (A) se encuentran los listados de extracción que contienen los posibles casos nuevos de cáncer y en el conjunto de datos (B) las bases de datos del sistema de información del RPCC. Primero se divide el conjunto de datos en grupos más pequeños para optimizar los apareamientos, luego se estandarizan y se indexan por bloques de similitud entre dos campos (nombres y fecha de nacimiento), por último se realiza una clasificación vectorial ponderada, donde se establece un umbral de similitud, el resultado son dos grupos de registros: los que se estiman como potencialmente iguales y los que se consideran como un posible apareamiento cuyo proceso continúa con una revisión manual, se evalúan los registros para ser apareados entre los dos conjuntos de datos ^16^.

### Procedimientos para el análisis de la incidencia y mortalidad: 

La Clasificación Internacional de Enfermedades (CIE-10) ^17^ es utilizada para la codificación del cáncer. Las principales localizaciones se definieron de acuerdo con las guías sugeridas por la IARC para el análisis de la información de incidencia; y por la OMS para agrupar el sitio primario del tumor y las causas de la muerte por cáncer ^18^^,^^19^. La estructura de la población por sexo y grupos quinquenales de edad para cada año calendario se obtuvo en el Departamento Administrativo Nacional de Estadística de Colombia (DANE) ^5^. Las tasas de incidencia y de mortalidad para toda la población se estandarizaron por edad (ASR, por sus siglas en inglés *Age-standardised rate*) mediante el método directo, utilizando como referencia la población estándar mundial ^20^^,^^21^. Las tasas globales y específicas por edad y sexo están expresadas por 100,000 personas-años. Las tendencias de las tasas de incidencia se analizaron durante diez quinquenios de 1962 a 2012; y las de la mortalidad durante seis quinquenios, desde 1984 hasta 2015. La medida de resumen de la tendencia de las tasas fue el porcentaje de cambio anual (APC, por sus siglas en inglés - *Annual Percent Change*), calculado por el método de mínimos cuadrados ponderados ^22^. Para algunas localizaciones y grupos de edad fue imposible estimar el APC porque en algunos años no hubo casos nuevos o muertes por cáncer en dichas categorías. 

### Procedimiento para el análisis de supervivencia

#### Criterios de selección

Se incluyeron para el análisis los datos individuales de 38,671 residentes permanentes de Cali durante el periodo 1995-2009, con edad entre 15 y 99 años, con diagnóstico de un primer tumor maligno invasivo en una de las siguientes catorce localizaciones definidas por la CIE-10 (WHO, 2012): Estómago (C16), colorectal (C18-C20), hígado (C22), pulmón (C34), melanoma (C43), mama (C50), cuello uterino (C53), ovario (C56), próstata (C61), tiroides (C73), linfoma Hodgkin (C81), linfoma no Hodgkin (C82-C85, C96), mieloma múltiple (C90), leucemia (C91-C95). Siguiendo las guías del estudio Concord-2 ^23^, los grupos de tumores sólidos fueron definidos por el sitio anatómico y las leucemias por su morfología. La codificación de la topografía y morfología se hizo con la Clasificación Internacional de Enfermedad para Oncología, tercera edición (CIE-O-3) ^11^. Se incluyeron todas las enfermedades malignas hematopoyéticas según el rango de códigos morfológicos de la CIE-O-3 desde 9,590 hasta 9,989. 

Se excluyeron del análisis de supervivencia: los tumores identificados como *in situ*, benignos o de comportamiento incierto, sujetos con edades desconocidas, los tumores detectados durante la necropsia, los casos en los que la única evidencia de cáncer fue el certificado de defunción, los síndromes mielodisplásicos y las neoplasias mieloproliferativas como la leucemia mieloide crónica. Las personas con cáncer de mama bilateral sincrónico fueron incluidas y tratadas como casos individuales para el análisis.

**Definición de evento, fecha inicial y final** La muerte por cualquier causa se consideró una falla. El tiempo de supervivencia de cada caso se determinó por la diferencia de tiempo (en días) entre la fecha de diagnóstico (fecha índice) y la fecha de la muerte, la fecha del último contacto, o la fecha de cierre del seguimiento, la cual fue definida como el 31 de diciembre de 2009. Para comparar el comportamiento de la supervivencia durante el tiempo analizado, el lapso de 15 años se dividió en tres: un primer periodo comprendido entre 1 de enero de 1995 y 31 de diciembre de 1999 que coincidió con la implementación de la reforma a la salud en Colombia; y los otros dos periodos; 2000-2004 y 2005-2009; después de la implementación y consolidación de la Ley 100. 

#### Seguimiento

Para actualizar el estado vital y la fecha del último contacto, se establecieron enlaces entre el sistema de información del RPCC y las siguientes bases de datos: a) mortalidad general de la Secretaría de Salud Pública Municipal de Cali, b) egresos hospitalarios de instituciones de nivel III; c) Sistema de Identificación de Potenciales Beneficiarios de Programas Sociales (SISBEN, 2016) y d) Empresas aseguradoras de salud privadas (2014). Proceso descrito en las Figuras 3 y 4.

### Plan de análisis 

La variable respuesta fue el tiempo transcurrido entre el diagnóstico de cáncer y la muerte de cada individuo. El tiempo máximo de observación para cada sujeto para que ocurra la falla fue cinco años. La variable de censura fue definida para las personas que no presentaron la falla dentro del periodo de estudio, y como mecanismos de censura se establecieron la pérdida durante el seguimiento y la finalización del estudio. Para el análisis fueron censuraron los tiempos de supervivencia mayores a cinco años, los tiempos posteriores a la pérdida durante el seguimiento y/o a 31-12-2009. 

Para los quinquenios 1995-1999 y 2000-2004 se realizó un análisis de cohorte porque todos los pacientes con diagnóstico de cáncer durante ese lapso tuvieron al menos cinco años de información disponible del seguimiento hasta el 31 diciembre 2009. Para el quinquenio 2005-2009, el monitoreo del análisis de supervivencia se llevó a cabo mediante un análisis de periodo ^24^, dado que no se tiene información completa de 5 años de seguimiento para todas las personas, como se presenta en la Figura 5. El número en cada celda indica el número mínimo de años de seguimiento completado por los pacientes al final de un año específico.

**Figura 5:** Registro Poblacional de Cáncer de Cali. Estructura de monitoreo para el análisis de supervivencia de los casos de cáncer en residentes permanentes de Cali durante el intervalo 1995-2009 con seguimiento hasta 2009-12-31. Se analizan tres quinquenios: 1995-1999, 2000-2004 y 2005-2009. Enfoque de cohorte entre 1995-2004 y de periodo entre 2005-2009. En celdas contiguas se describe el seguimiento completo de un intervalo. Ejemplo: Todos los pacientes con diagnóstico en 1995 completan un año de seguimiento en 1996, exceptuando los diagnosticados el 1995-01-01.

Para las estimaciones de la supervivencia neta a 5 años se utilizó el estimador de Pohar-Perme ^25^. Las tablas de vidas de la población general de Cali en edades simples, por sexo y para cada año calendario desde 1995 a 2010, fueron hechas para el estudio Concord-2 ^23^. Las estimaciones de supervivencia neta a 5 años se estandarizaron por edad para lograr comparaciones a través del tiempo o con diferentes poblaciones de cáncer y con diferentes distribuciones de edad. Se tuvo en cuenta los tres grandes grupos de localizaciones de cáncer con patrones de incidencia similares por edad y se implementaron los ponderadores de la Norma Internacional de la Supervivencia del *Cancer, International Cancer Survival Standar ICSS* ^26^ (Tabla 1). 

**Tabla 1:** Pesos para estandarizar por edad para las estimaciones de supervivencia neta según el tipo de cáncer. (ICSS por su sigla en inglés *International Cancer Survival Standard*)

ICSS-1		ICSS-1*		ICSS-2		ICSS-3	
Grupo edad	Pesos	Grupo Edad	Pesos	Grupo Edad	Pesos	Grupo Edad	Pesos
15-44	0.07	15-54	0.19	15-44	0.28	15-44	0.6
45-54	0.12	55-64	0.23	45-54	0.17	45-54	0.1
55-64	0.23	65-74	0.29	55-64	0.21	55-64	0.1
65-74	0.29	75-84	0.23	65-74	0.20	65-74	0.1
75+	0.29	85+	0.06	75+	0.14	75+	0.1
Total	1		1		1		1

ICSS-1*: Próstata (C61)

**ICSS-1:** Estómago (C16), colorrectal (C18-C20), hígado (C22), pulmón (C34), mama (C50), ovario (C53), linfoma no Hodgkin (C82-C85, C96), mieloma múltiple (C90), leucemia (C91-C95)

**ICSS-2:** Melanoma (C43), cuello-uterino (C53), tiroides (C73)

**ICSS-3:** Linfoma Hodgkin (C81)

### Evaluación de la exhaustividad con el método del certificado de defunción

Para comprobar la exhaustividad se utilizó el método del certificado de defunción ^27^. El principio se ilustra en la Figura 6. Los certificados individuales de la mortalidad general por todas las causas se reciben anualmente en un archivo estructurado en formato digital con información de las causas de muerte en texto y la causa básica codificada con CIE-10 ^17^. Se revisa las causas de muerte que están texto para detectar los casos con mención de cáncer que no fueron codificados como cáncer en la causa básica; y se crea una variable para identificar los casos de cáncer (CIE-10: C00-C97; D05-D06, D32-D33, D45-D46, D47.1, D47.3) y los residentes de Cali. El apareamiento inicial con la base de datos del RPCC permite identificar los casos prevalentes que han fallecido, se actualiza el estado vital y la fecha de muerte. Los casos nuevos notificados anualmente mediante el certificado de defunción se incluyen en la base de datos del RPCC y se identifican en una variable como (DCN, por su sigla en inglés). Estos casos quedan en espera para actualizar la base más válida para el diagnóstico de cáncer. Cuando los recolectores del RPCC obtienen la información de la biopsia, el aspirado de medula ósea, o la citometría de flujo; se actualiza el método de diagnóstico, de certificado de defunción a diagnóstico por morfología. La búsqueda activa y continua de los casos excluye algunos de los casos de mortalidad que no son cáncer; y actualiza una vez más el método diagnóstico, de certificado de defunción a diagnóstico por clínica o por imágenes. Finalmente, queda un remanente de casos cuya única evidencia de cáncer es el certificado de defunción (DCO, por su sigla en inglés). La proporción de casos no registrados que permanecieron vivos se estimó con la proporción de casos iniciados por el certificado de defunción (DCI) y la razón mortalidad:incidencia (M:I). Exhaustividad = $1-DCI\;*(\frac{1}{M:I})/(1-DCI)$

**Figura 6:** Comprobación de la exhaustividad. El apareamiento anual entre la base de datos de mortalidad y el registro de cáncer permite identificar los casos nuevos notificados mediante el certificado de defunción, (DCN por su sigla en inglés).

La búsqueda activa y continua de los casos actualiza la base más válida para el diagnóstico y excluye algunos de los casos de mortalidad que no son cáncer (Rastreo no cáncer). El remante son los casos iniciados por el certificado de defunción (DCI por su sigla en inglés), que no habrían sido detectados por otra vía. Después de todas las maniobras de rastreo, queda un residuo de casos para los cuales la única evidencia de cáncer fue el certificado de defunción (DCO por su sigla en inglés).

## Resultados

### Indicadores de calidad de la información de incidencia

Los principales indicadores de calidad para algunas localizaciones seleccionadas de cáncer se presentan en la Tabla 2. La edad fue conocida en 99.4% de los pacientes. La razón mortalidad incidencia mostró valores consistentes con excepción de hígado (1.43) y pulmón (1.02). En estas localizaciones, el número de defunciones fue mayor que el número de casos detectados en vida.

**Tabla 2:** Cali, Colombia. Indicadores de la calidad de la información de incidencia para ambos sexos durante el periodo 2008-2012

Localización	n	Age (%)	M/I	1-NS	MV (%)	DCO (%)	Código CIE 10
Estómago	1,810	99.7	0.78	0.83	85.1	3.0	C16
Colorrectal	1,827	99.6	0.55	0.59	89.8	1.4	C18-20
Hígado	467	99.8	1.43	0.95	68.3	4.5	C22
Pulmón	1,316	99.9	1.02	0.90	66.4	6.0	C33-34, C38-39
Melanoma de piel	324	98.2	0.40	0.34	100.0	0.0	C43
Mama*	2,998	99.5	0.33	0.26	97.0	0.3	C50
Cérvix uterino	1,037	99.5	0.45	0.42	94.4	1.2	C53
Ovario	513	100.0	0.59	0.66	87.1	0.6	C56
Próstata	2,937	99.2	0.32	0.17	89.2	2.0	C61
Tiroides	1,066	98.0	0.07	0.11	97.9	0.3	C73
Enfermedad de Hodgkin	154	100.0	0.20	0.36	100.0	0.0	C81
Linfoma no Hodgkin	1,013	99.9	0.35	0.57	99.6	0.0	C82-C85, C96
Mieloma múltiple	298	99.7	0.55	0.77	99.7	0.0	C90
Leucemia	867	99.5	0.67	0.72	100.0	0.0	C91-C95
Todos los sitios	23,046	99.4	0.51		88.5	1.7	

M/I: Razón mortalidad:incidencia

MV: Proporción de casos verificados microscópicamente

DCO: Única evidencia certificado de defunción

NS: Supervivencia neta

* Se incluyen 26 casos de mama en hombres

El porcentaje de casos con verificación morfológico (VM) -histología, citología, aspirado de médula ósea y citometría de flujo-, para todas las localizaciones de cáncer fue de 88.5% rango de 85-100%, excepto en hígado (68.3%) y pulmón (66.4%). En los pacientes con leucemia, linfoma de Hodgkin y melanoma la VM fue del 100%. 

El porcentaje de casos con certificado de defunción como única evidencia del diagnóstico de cáncer (DCO) varió entre 0-3%, excepto en hígado (4.5%) y en pulmón (6.0%). En general, las localizaciones más frecuentes presentan un bajo porcentaje de casos obtenidos solamente a través del certificado de defunción. Otro indicador de calidad que también suele considerarse es la proporción de los casos de cáncer que fue codificada como de sitio mal definido. Entre los años 2008-2012 estos tumores han representado el 4,6% de los casos nuevos de cáncer en los hombres y el 5.4% en las mujeres. 

### Indicadores de calidad de la información de supervivencia

Durante el periodo 1995-2009 se registraron 40,354 casos de los canceres seleccionados, 1.73% ocurrieron en pacientes menores de 15 años. En 2.4% no hubo información de edad y se excluyeron del análisis. Todos los pacientes tuvieron seguimiento y se censuraron el 13.2% de las observaciones; esta proporción fue mayor en cáncer de cerebro, melanoma, colorrectal y ovario. En los cánceres con pobre supervivencia: estómago, pulmón, hígado y páncreas; la proporción de censura fue menor al 10%. En los cánceres más frecuentes el porcentaje de censura fue 10.1%, 11.5% y 16.4%; para mama, próstata y cérvix, respectivamente. En 15.3% la fecha de muerte y la fecha de incidencia fue la misma. 

### Indicadores de calidad de la certificación de la mortalidad por cáncer

La mortalidad por cáncer representó el 18.0% (23,793/132,397) de la mortalidad general ocurrida en la ciudad durante el periodo 2006-2015. El 0.8% de los casos no fue codificado como cáncer en la causa básica. Todas las defunciones fueron certificadas por un médico; la proporción de sitio mal definido (C76-C80, C97) fue 5.3% y la de cáncer de útero no especificado (C55) fue 0.5%. Solo 4(0.02%) de los certificados de defunción no tuvieron información de la edad. El 94.2% de las muertes por cáncer fueron bien certificadas. 

Todos los pacientes muertos por cáncer durante el periodo 2008-2012 se encontraron en la base de datos del registro de cáncer. Para los sitios reconocidos de metástasis; hígado, pulmón, hueso y cerebro; se comparó el código de la CIE-10 ^17^ del certificado de defunción con el código topográfico de la CIE-0-3 ^11^ asignado por el registro de cáncer. La Tabla 3 muestra la concordancia de los dos sistemas para asignar el código para cada una de las localizaciones descritas. El 45% de las defunciones codificadas como cáncer de hígado en el certificado de defunción correspondían a metástasis. En el RPCC había evidencias (verificación morfológica y clínica) de haber realizado el diagnóstico de cáncer en vida del paciente en un sitio primario diferente. Esta proporción alcanzó valores de 46%, 15% y 10% para las localizaciones de huesos, pulmón y SNC. 

**Tabla 3:** Cali, Colombia. Concordancia de la codificación para algunos sitios seleccionados entre el Registro Poblacional de Cáncer de Cali y la Secretaría de Salud Pública Municipal. 2008-2012.

Fuente información			Registro de cáncer			
Secretaría de Salud Pública Municipal	Localización		Hígado				
			Si	No	Kappa	IC 95%	
	Hígado	Si	341	284	0.6382	0.60	0.68
		No	3	1,819			
	Pulmón		Pulmón				
		Si	980	250	0.7902	0.75	0.83
		No	7	1,210			
	Hueso		Hueso				
		Si	50	43	0.6711	0.63	0.71
		No	4	2,350			
	SNC		SNC				
		Si	430	69	0.8985	0.50	1.29
		No	8	1,940			

### Evaluación de la exhaustividad con el método del certificado de defunción

El 54% de las personas que murieron por cáncer en Cali durante el periodo 2008-2012 ya estaban incluidas en la base de datos del RPCC. Los casos nuevos notificados mediante el certificado de defunción (DCN) correspondieron al 46% de las defunciones del periodo. La proporción fue más alta en los canceres con alta letalidad como estómago (45%); y más baja, en los pacientes con cáncer de mama (12%), cuello uterino (18%), próstata (14%) y cáncer infantil (23%), Tabla 4. El índice de exhaustividad fue mayor a 90%, excepto en los casos de cáncer de próstata.

**Tabla 4:** Registro Poblacional de Cáncer de Cali, Colombia. Porcentaje de casos registrados como DCN, DCO, y razón mortalidad:incidencia; en localizaciones seleccionadas. Periodo 2008-2012.

Localización	DCN	DCN/M	DCO	DCO/M	Incidencia	Mortalidad	M:I	DCI	DCI/M	**Exhaustividad**	
	**n**	**%**	**n**	**%**	**n**	**n**		**n**	**%**
Todas	5,327	0.46	371	0.03	23,046	11,664	0.51	1,403	0.12	0.87
Estómago	625	0.45	53	0.04	1,810	1,374	0.72	99	0.07	0.97
Colorrectal	332	0.34	25	0.03	1,827	987	0.53	68	0.07	0.94
Mama	115	0.12	7	0.01	2,972	941	0.32	32	0.03	0.92
Cérvix	81	0.18	11	0.02	1,037	462	0.45	16	0.03	0.96
Próstata	126	0.14	54	0.06	2,937	913	0.31	103	0.11	0.72
Infantil <15	38	0.23	1	0.01	402	167	0.42	3	0.018	0.97

I: Incidencia; M: Mortalidad

DCN: Casos nuevos notificados anualmente al RPCC mediante el certificado de defunción.

DCI: DCN - casos excluidos de mortalidad que no son cáncer

DCO: Casos nuevos en el RPCC cuya única evidencia de cáncer es el certificado de defunción.

Exhaustividad: (1-DCI*(1/M:I))/(1-DCI

### Encuesta periódica a los servicios oncológicos

La última encuesta realizada en 2,014 tuvo un costo de US$23,300, participaron 19 estudiantes, cada uno recibió una bonificación, subsidio de transporte y un pago por encuesta realizada. Durante las ocho semanas se visitaron 107 servicios oncológicos, ubicados en centros médicos (64), clínicas particulares (36) y consultorios particulares (5). Se obtuvo información que permitió identificar 2,215 casos nuevos de cáncer (27.8%) y actualizar 5,750 casos (72.2%) que ya estaban en la base de datos del RPCC.

## Discusión 

El registro de cáncer de Cali es el único entre los países de bajos y medianos ingresos que ha reportado con exactitud la situación del cáncer de forma continua durante el último medio siglo. La información es de alta calidad y ha sido incluida en todos los once volúmenes de *Cancer Incidence in Five Continents*(CI5)^21^^,^^28^^-^^37^. Durante cuarenta años el (RPC-Cali) fue la única fuente de información válida de la incidencia de cáncer en Colombia ^2^. Para aumentar la cobertura, el Instituto Nacional de Cáncer de Colombia (INC-Col) con la asesoría de la Universidad del Valle, impulsó durante la primera década del siglo XXI la creación de RPCs en regiones estratégicas del país. Gracias a este esfuerzo la información de incidencia de las ciudades colombianas de Pasto, Manizales y Bucaramanga fue sumada a la de Cali y publicada en el 2012 en CI5 ^36^, y los cuatro RPC colombianos participaron en el estudio CONCORD ^23^, el programa global para la vigilancia mundial de la supervivencia al cáncer, dirigido por la Escuela de Higiene y Medicina Tropical de Londres. En la actualidad, el RPC-Cali participa en SURVCAN-3, una iniciativa de la IARC para producir estadísticas de supervivencia confiables y comparables para países en transición. Por la gran fortaleza del Registro de Cáncer, Cali es la primera ciudad del mundo en implementar la iniciativa “C/Can 2025: Desafío de Ciudades Contra el Cáncer”; una iniciativa de la Unión Internacional Control del Cáncer (UICC) que busca aumentar la cobertura y la calidad de la atención oncológica en las ciudades de más de un millón de habitantes de los países de bajos y medianos ingresos.

### Factores del éxito del RPCC

Varios factores han contribuido a la permanencia y continuidad en el tiempo del Registro de Cáncer de Cali. El RPCC tiene definiciones y procedimientos estandarizados para la recolección, análisis, almacenamiento, validación y divulgación de la información. La Universidad del Valle ha sido la principal fuente de recursos financieros y técnicos. Los cuatro directores que ha tenido el RPCC en los 55 años de operación han sido académicos e investigadores del Departamento de Patología, encargados de coordinar un talento humano capacitado que pertenece a la planta de la Universidad del Valle. El RPCC está constituido como grupo de investigación y escalafonado en la cima del sistema de investigación colombiano. Con proyectos específicos resuelve los problemas epidemiológicos y complementa las actividades de recolección de información. El costo total por caso en el RPCC fue US$82, que incluyó US$25 para actividades de costos fijos, US$43 para actividades centrales de costo variable y US$14 para otras actividades ^38^. 

 El RPCC tiene reconocimiento social en la ciudad, facilitando así el proceso de recopilación de datos que se hace de manera pasiva y activa en las diferentes fuentes de información. La infraestructura para la atención oncológica en Cali, cuenta con 165 servicios oncológicos habilitados ^8^ para ofrecer diagnóstico correcto y tratamiento adecuado a 9000 pacientes anuales ^39^ Desde su fundación en 1962, el RPCC limitó el área de registro al casco urbano de Cali y elaboró una definición clara de “caso”, incluyendo solo los casos nuevos de cáncer diagnosticados en los residentes permanente de la ciudad; y excluyendo los casos de pacientes remitidos a la ciudad para procedimientos de diagnóstico y/o tratamiento. Para la estimación de las tasas y para construir las tablas de vida para el estudio de la supervivencia, se requiere denominadores fiables basados en los censos y proyecciones de población. El Departamento Nacional de Estadística de Colombia ha facilitado la estructura de la población desagregadas según años calendario, sexo, grupos de edad y total edades simples para el periodo 1962-2015 

### **Normatividad para la notificación del cáncer en Colombia**. 

El gobierno colombiano posicionó al cáncer como un problema de salud pública y estableció las acciones para la atención integral para reducir la morbimortalidad por este grupo de enfermedades y mejorar la calidad de vida de los pacientes oncológicos. Se implementaron los mecanismos de vigilancia y control y se organizó el Sistema Nacional de Información de Cáncer. El modelo de atención para estas enfermedades está definido en el Plan Decenal para el Control del Cáncer en Colombia 2012-2021 ^40^, y el eje de este plan estratégico es la vigilancia, los análisis situacionales y la investigación. El Ministerio de Salud y Protección de Colombia (MSPS-Colombia) reglamentó los datos básicos que deben reportar las aseguradoras de salud y las entidades sanitarias sobre los servicios oncológicos prestados, sean estos de promoción, prevención, diagnóstico, tratamiento o rehabilitación (RIPS). Para la gestión de la información en salud publica reglamentó el Sistema de Vigilancia en Salud Pública (SIVIGILA) para la vigilancia del cáncer de mama, cáncer de cuello uterino y cáncer infantil; el objetivo es determinar la oportunidad en el inicio del tratamiento de los casos confirmados y estimar la frecuencia de los casos que se detectan en los diferentes estadios. Desde el año 2014, las aseguradoras de salud (EPS) deben reportar la información de cáncer a la Cuenta de Alto Costo (CAC), un organismo no gubernamental que fue creado para orientar la gestión del riesgo en salud y garantizar el gerenciamiento de la enfermedad de las personas afectadas. 

### Fortalezas del RPCC: Índices de calidad durante el periodo 2008-2012 

El valor de un registro de cáncer depende enormemente de la calidad de los datos y de los procedimientos de control de calidad vigentes ^4^. El RPC-Cali tiene en cuenta cuatro dimensiones para los indicadores de calidad de los datos recolectados: comparabilidad, validez, oportunidad y exhaustividad. 

#### Comparabilidad

El RPCC utiliza métodos estándar para que la información sea comparable con otras regiones del país y del mundo. En los adultos las neoplasias se codifican con la CIEO-3 ^11^ y en los niños con la ICCC-3 ^12^. Para la fecha de incidencia se siguen las guías de la EUROPEA ^13^ y para la clasificación de los tumores primarios múltiples las guías de IARC/IACR ^14^

#### Validez

Las principales y más confiables fuentes de datos para el registro de cáncer son los informes de histopatología; pero no son suficientes para garantizar la exhaustividad. En tumores poco accesibles tales como los del SNC, páncreas, pulmón, retroperitoneo y otros; la base del diagnóstico puede ser los estudios de imágenes, la exploración clínica y los DCO. 

El porcentaje de casos del RPC-Cali con un diagnostico morfológicamente verificado (MV%) fue 88.5%, similar a otros RPC-colombianos y RPC-Latinoamericanos; e inferior a la mayoría de RPC-europeos y RPCC-norteamericanos (90%-95%) ^37^. África tiene los dos contrastes (53.9% Uganda: Kyandono Country; 97.8% Algeria: Sétif) ^37^. En los países de bajos y medianos ingresos, una gran proporción de los casos diagnosticados a través del servicio de patología puede sugerir deficiencias en la búsqueda de casos y, por lo tanto, evidencias de un registro incompleto. 

En el RPC-Cali, el porcentaje de casos conocidos sólo por certificado de defunción (DCO%) fue 1.7%; el más bajo de todos los RPC-latinoamericanos; y similar a la mayoría de RPC-norteamericanos y RPC-europeos ^37^. Algunos RPC-africanos y RPC-latinoamericanos tienen DCO% mayor al 10%; indica deficiente detección del caso y mala calidad, porque los certificados de defunción no proporcionan información sobre la morfología del tumor. Una alta proporción de casos nuevos de cáncer con base en un diagnóstico clínicos tiene igual interpretación. 

#### Exhaustividad

Las tasas de incidencia han sido estables en el tiempo y los valores esperados son comparables con los reportados por los registros de cáncer que atienden a poblaciones similares como Quito (192.8 personas-año y 198.9 personas-año en hombres y mujeres, respectivamente) y Costa Rica (173.9 personas-año y 167.0 personas-año en hombres y mujeres, respectivamente) ^37^. El trabajo colaborativo con la SSPM de Cali facilita el acceso a la información de la mortalidad general y por cáncer; y permite tener una fuente independiente de verificación de los casos nuevos de cáncer. El 94.2% de las muertes por cáncer fueron bien certificadas. La razón M:I para todas las localizaciones de cáncer en Cali durante el periodo 2008-2012, fue de 51%; similar a la de otros RPC-latinoamericanos (rango, 38.3% a 68%) ^36^ y mayor a la reportada por Estados Unidos (34.8% en hombres; y 36% en mujeres) ^36^ a través del SEER (*Surveillance, Epidemiology, and End Results Program*). En muchos países de Latinoamérica la razón M:I es mayor a 1 en tumores con alta letalidad como páncreas, hígado, esófago, por falta de exhaustividad y/o falta de diagnóstico cuando el paciente estaba vivo. 

El índice de exhaustividad mediante el método de los certificados de defunción (IE-CD) fue 87%; y en los canceres priorizados por el PNDC fue mayor a 90%, excepto en los casos de cáncer de próstata (72%). Este índice del RPC-Cali es más alto que el reportado por otros registros de cáncer internacionales (82.8% en Japón, Miyagi; 80.4% en Alemania, Munster y 65.6% en Reino Unido) ^41^. El método depende de la disponibilidad de certificados de una calidad relativamente buena, que mencionen la causa de defunción (de manera completa y exacta) en el área cubierta por el registro de cáncer. Este método no ha sido aplicado en otros RPC-latinoamericanos. 

#### Oportunidad

Las estadísticas de la situación del cáncer en Cali son de acceso público en los 36 meses siguientes al año de diagnóstico y se encuentran disponibles en el portal del RPPC http://rpcc.univalle.edu.co. La información organizada en páginas web describe 50 años de incidencia (1962-2012), 30 años de mortalidad (1984-2014) y 15 años de supervivencia (1995-2009). 

#### Limitaciones 

Los datos de cada servicio en cada institución se manejan de manera autónoma e independiente. La información cruza cada proceso fragmentada y en diferentes plataformas, generando la duplicación de datos, dificultades de transferencia y un decremento en la calidad e integridad de la información. Los servicios oncológicos colombianos hacen notificación periódica a diferentes dependencias del ministerio de salud (SIVIGILA, RIPS, CAC). Estos sistemas heredados, en su mayoría son aplicaciones locales que carecen de interoperabilidad para una adecuada gestión de los datos. Las instituciones empiezan a percibir a la notificación como una carga y relegan a un segundo plano la transferencia de datos al registro de cáncer. Esta complejidad es un factor de riesgo para garantizar la exhaustividad en la recolección de información. En consecuencia, hay grandes posibilidades de subestimar el riesgo de cáncer en la población. Es urgente modificar la normatividad vigente del Ministerio de Salud debe modificarse para que los RPC-colombianos se incorporen al sistema de información de cáncer con una adecuada asignación presupuestal. 

### Retos futuros

Es prioritario la implementación de estándares y mecanismo de transferencia, flujos compartidos de información y adopción de herramientas que permitan comunicar lo diferentes sistemas de información en la ciudad de Cali. Así mismo fijar políticas públicas que faciliten la ejecución de estas soluciones y. Luego, es imprescindible la creación de una bodega de datos interinstitucional con el fin de proporcionar soportes claves para la toma de decisiones tanto públicas a nivel poblacional como administrativas a nivel clínico. El principal objetivo de esta implementación es garantizar información de calidad para la gestión del conocimiento.
