# Supplementary material for: Cali Cancer Registry Methods
Source: Colomb Med (Cali). 2018 Jun 30;49(2):188. doi: 10.25100/cm.v49i2.4056 (PMC6084916; doi:10.25100/cm.v49i2.4056)
Supplement: Translation

# Translation: Cali Cancer Registry Methods

Este artículo de Garcia et al, aparece como autor de correspondencia: “Luz Stella Garcia, Registro Poblacional de Cancer de Cali. Calle 4B 31-00 Oficina 4003, Edificio 116, Cali, Colombia. E-mail: luz.garcia@correounivalle. edu.co”, el autor de correspondencia correcto es “Luis Eduardo Bravo, Director Registro Poblacional de Cáncer de Cali. Calle 4B 31-00 Oficina 4003, Edificio 116, Cali, Colombia. E-mail: luis.bravo@correounivalle.edu.co”
